# Synthesis of Ribose-Coated Copper-Based Metal–Organic Framework for Enhanced Antibacterial Potential of Chloramphenicol against Multi-Drug Resistant Bacteria

**DOI:** 10.3390/antibiotics10121469

**Published:** 2021-11-29

**Authors:** Haseena, Adnan Khan, Iqra Ghaffar, Roua S. Baty, Mohamed M. Abdel-Daim, Shahida M. Habib, Tasmina Kanwal, Muhammad Raza Shah

**Affiliations:** 1 Institute of Chemical Sciences, University of Peshawar, Peshawar 25120, Pakistan; haseenafarid5@gmail.com (H.); adnankhan@uop.edu.pk (A.K.); 2 International Centre for Chemical and Biological Sciences, Research Institute of Chemistry, University of Karachi, Karachi 74200, Pakistan; iqraghaffar47@yahoo.com (I.G.); shahidahabib92@gmail.com (S.M.H.); tasmina93.kanwal@gmail.com (T.K.); 3 Department of Biotechnology, College of Science, Taif University, P.O. Box 11099, Taif 21944, Saudi Arabia; rsbaty@tu.edu.sa; 4 Pharmacology Department, Faculty of Veterinary Medicine, Suez Canal University, Ismailia 41522, Egypt; abdeldaim.m@vet.suez.edu.eg

**Keywords:** multi-drug resistance, metal–organic framework, chloramphenicol, biofilm, antibiotic

## Abstract

The rise in bacterial resistance to currently used antibiotics is the main focus of medical researchers. Bacterial multidrug resistance (MDR) is a major threat to humans, as it is linked to greater rates of chronic disease and mortality. Hence, there is an urgent need for developing effective strategies to overcome the bacterial MDR. Metal–organic frameworks (MOFs) are a new class of porous crystalline materials made up of metal ions and organic ligands that can vary their pore size and structure to better encapsulate drug candidates. This study reports the synthesis of ribose-coated Cu-MOFs for enhanced bactericidal activity of chloramphenicol (CHL) against *Escherichia coli* (resistant and sensitive) and MDR *Pseudomonas aeruginosa*. The synthesized Cu-MOFs were characterized with DLS, FT-IR, powder X-ray diffraction, scanning electron microscope, and atomic force microscope. They were further investigated for their efficacy against selected bacterial strains. The synthesized ribose-coated Cu-MOFs were observed as spherical shape structure with the particle size of 562.84 ± 13.42 nm. CHL caused the increased inhibition of *E. coli* and MDR *P. aeruginosa* with significantly reduced MIC and MBIC values after being encapsulated in ribose-coated Cu-MOFs. The morphological analysis of the bacterial strains treated with ribose-coated CHL-Cu-MOFs showed the complete morphological distortion of both *E. coli* and MDR *P. aeruginosa.* Based on the results of the study, it can be suggested that ribose-coated Cu-MOFs may be an effective alternate candidate to overcome the MDR and provide new perspective for the treatment of MDR bacterial infections.

## 1. Introduction

Chloramphenicol (CHL) is a broad-spectrum antibiotic having remarkable efficiency against Gram-positive and Gram-negative bacteria and often administrated to human and animals to treat bacterial infections [1]. The antibacterial activity of CHL has been reported against various pathogens including *Neisseria meningitides*, *Streptococcus pneumoniae*, and *Haemophilus influenza* [2]. CHL inhibits the bacterial growth by inhibiting the activity of peptidyl transferase via binding with 50S subunit of bacterial ribosome which in turn inhibits the elongation of protein-chain [3]. CHL also possess efficacy against *Enterococcus faecium* and can be used for the treatment of vancomycin-resistant *Enterococcus*. Furthermore, CHL is also reported to be used as first-choice treatment for staphylococcal brain abscesses because of having promising penetration across blood–brain barrier [4].

Metal–organic frameworks (MOFs) are a new class of porous crystalline materials formed by the linkage of metal ions and organic ligands which give rise to extensively rigid open-network structure. These crystalline materials have attracted the great interest of researchers and are widely used as an efficient nano-carrier [5]. These crystalline materials having capability to change their pore size, structure and provide enhanced encapsulation of desired drug candidate. These features make the MOFs as a good drug delivery vehicle [6]. MOFs have been used as catalyst, chemical sensor, and drug delivery carrier with the greatest application in gas storage [7,8,9]. Although various drug delivery systems have been reported, MOFs are preferred among all because of their remarkable advantages which includes the structure flexibility that control the architecture of these substances, and they are responsible for diverse shapes, and unique physicochemical characteristics, composition, size, and post-synthesis modification [10]. For the antibacterial agents, MOFs can be served as a reservoir due to their composition, construction, and internal surface volume. These properties are advantageous to use MOFs as a new high-performance material with antibacterial properties [11]. The physicochemical properties of MOFs can be changed significantly by the manipulation in the metal ion centers or organic linkers but the slight modification in synthetic condition is also capable of regulating the size and shape of MOFs. It is important to control the particle size because of the fact that larger surface area is responsible for enhanced bactericidal potential of nanocarriers [12]. Lee and coworkers previously developed a series of robust MOFs with 3D frameworks and high surface area-to-volume ratios that exhibited superior antibacterial activity [13,14,15].

Copper has the ability to prevent the biofilm formation with low or negligible toxicity [16]. In the old era, copper was used for the sterilization of wounds and drinking water [17]. Furthermore, copper is also known to show antibacterial potential by following various mechanisms which includes accumulation of the cell wall which results in decreased permeability, intracellular leakage, cellular internalization, damage of DNA structure, and critical enzymes [18]. Hence, it would be a unique strategy to design copper containing MOFs for the development of effective antibacterial materials.

The increase of bacterial resistance against the conventional antibiotics is the topmost concern for the researcher of the medical field, and they are in continuous search for an effective alternative which can overcome the bacterial resistance with no or negligible side effects. The antibiotic resistance enhances the potential of bacteria to grow in the presence of one or more antibiotics [19]. Multi-drug resistance of bacteria is a significant danger to human civilization and it progresses at a faster speed owing to inappropriate and frequent use of antibiotics [20,21]. Bacterial infections are currently causing increased rates of mortality and chronic infections. Antibiotics are extensively prescribed and used to treat bacterial infections because they have superior clinical results and are less expensive [22]. However, in the future, a shortage of powerful antibiotics will make common infections such as bacterial pneumonia, as well as complex treatments such as open-heart surgery, far more risky and life-threatening [23]. Although resistance is multifactorial, one promiscuous mechanism spanning multiple antibiotic classes is the expression of so-called resistance-nodulation-division (RND) superfamily exporters, which mediate active efflux of small molecules, including various antibiotics, from the periplasm and inner membrane to the extracellular environment [24]. RND efflux system is comprised of a proton-motive force-driven inner membrane pump, a periplasmic adapter protein, and an outer membrane channel [25]. Multiple RND efflux systems exist in *Escherichia coli*, each with its own set of pumps and adaptor proteins, but they all rely on TolC, an outer membrane efflux protein. *Pseudomonas aeruginosa* has 18 different RND efflux systems, and the major outer membrane efflux protein OprM is required for antibiotic resistance under standard conditions, though overexpression of OpmJ or OmpH can replace OprM [26]. Upregulation of RND efflux systems increases antibiotic resistance in laboratory strains of various Gram-negative bacterial pathogens [24].

There is another mode of antibacterial tolerant showed by various pathogenic bacteria called biofilm. Biofilms are basically sessile communities of microbes formed on the various biotic and abiotic surfaces by producing an extracellular polymeric matrix [27]. Pathogenic bacterial biofilms provide resistance to antibiotics and are easily infective, causing severe damage to the surface to which they are attached as well as disrupting the flow systems [28]. The most common Gram-negative biofilm forming pathogens associated with medical devices are *Pseudomonas aeruginosa* and Escherichia coli [29]. *Escherichia coli* is the bacteria that is most frequently implicated in urinary catheter-related infections, accounting for 50% of all infections of this nature [30]. When *Pseudomonas aeruginosa* colonizes the lungs of cystic fibrosis patients, it forms thick antibiotic-resistant biofilms that also protect the infected patient from host immune defenses, lowering the patient’s long-term prognosis [31]. Generally, for the communication in the biofilm, bacteria use a quorum sensing (QS) system which is actually a molecule known as autoinducer-2 (AI-2) [32]. In this circumstance, QS inhibitors (QSIs) are used which significantly prevent the formation of biofilm and are also capable of decreasing their virulence within the biofilm. Hence, they are ideal to resist to pathogenic biofilm formation and provide the significant control of bacterial infection [33]. Ribose is considered as an ideal QSI because it is non-toxic and shares structural similarity with AI-2 [34]. This resemblance creates the competing behavior between AI-2 and ribose. Thus, ribose can be used as a QSI for the inhibition of biofilm formation of various pathogenic bacteria [35].

Another important molecule, carbohydrates, plays an important role as it is significantly involved in various biological processes such as the development and differentiation of living organisms and pathological processes [36]. It is present in both eukaryotic and bacterial cell in the form of glycans, proteoglycans, glycoproteins, or glycolipids, and performs the function of cell–cell interaction and communication, and signal transduction. Furthermore, it is critically involved in cell growth and various immune responses [37]. There are various pathogens with the capability of binding with cell surface carbohydrate and cause infection [38]. Generally, carbohydrates having a great influence on the cell surface adhesion process either via carbohydrate–carbohydrate or carbohydrate–receptor (lectin) interactions [39,40]. Lectins are the proteins that are present on the bacterial surface which are capable of interacting with host carbohydrate and causing infection.

Based on this fact, here we reported ribose-coated Cu-MOFs to enhance the antibacterial activity of CHL against *E. coli* (sensitive and resistant) strains and *P. aeruginosa.* Ribose coating on the Cu-MOFs helps to interact with lectins, which eventually promote the interaction of CHL with bacterial cell and leads to enhance bactericidal potential of CHL.

## 2. Experimental

### 2.1. Material

Copper (II) sulfate (CuSO_4_·7H_2_O, 99%) was purchased from Merck Millipore, 2-aminoterephthalic acid (NH_2_-BDC) was purchased from Shanghai Macklin Biochemical Co. Ltd. (Shanghai, China), and polyvinyl pyrrolidone (PVP), 5-diphenyltetrazolium bromide (MTT), chitosan, and poly-lysine were purchase form Sigma Aldrich (Darmstadt, Germany). Mueller Hinton broth and Tryptic soya agar were obtained from Oxoid, UK. Chloramphenicol was purchased from a local pharmaceuticals company. All solvents used in this study were of analytical grade and utilized without any purification.

### 2.2. Synthesis of Cu-MOFs

**Scheme 1:**
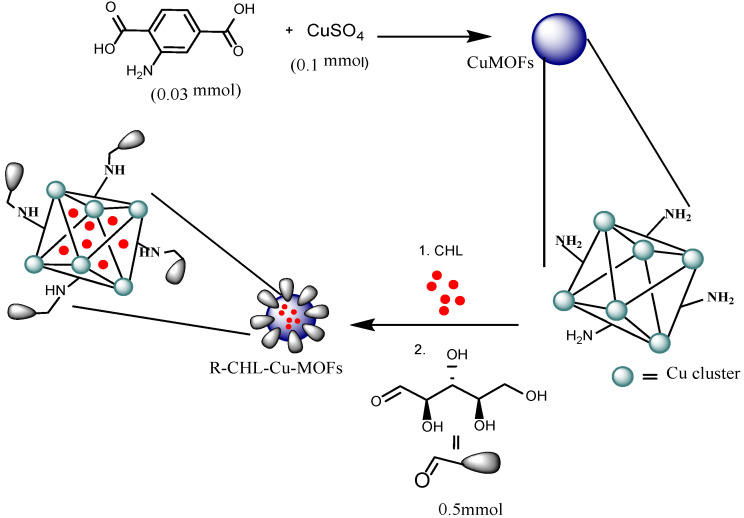
Schematic representation for the synthesis of R-CHL-Cu-MOFs.

The synthesis of Cu-MOFs was accomplished according to the previously reported protocol with slight modification (Scheme 1) [41]. CuSO_4_·7H_2_O (0.1 mmol) and 2-aminoterephthalic acid (0.03 mmol) was dissolved in 4 mL of DMF. In another flask, 0.20 g of PVP was dissolved in the mixed solvent system of DMF and ethanol (1:1 *v*/*v*). The resulting mixture was added to the above solution and sonicated for 30 min and kept at 110 °C for 8 h in hot air sterilizer. The resulting precipitate was then separated by centrifugation at 8000 G for 15 min and washed with DMF and ethanol to remove the unreacted substances and dried in vacuum oven [42].

### 2.3. Encapsulation of Chloramphenicol in Cu-MOFs

For the encapsulation of CHL in to the CuMOFs, an aqueous mixture of CHL (20 mg) and Cu-MOFs (10 mg) was stirred for 24 h at 200 G. Drug-loaded Cu-MOFs were separated by centrifugation at 10,000 G for 15 min. The drug loading efficiency (DLE) of Cu-MOFs were determined through UV-Vis spectrophotometer (UV-240, Shimadzu, Kyoto, Japan). Briefly, a specific amount of CHL-Cu-MOFs was dispersed in water and sonicated for 30 min. The mixture was then centrifuged at 10,000 G for 20 min. Supernatant was collected and analyzed for CHL. The drug was detected at 278 nm and %DLE was determined by using Equation (1) [43].

(1) $$\%DLE=\frac{amount\;of\;CHL\;in\;Cu-MOFs}{amount\;of\;CHL\;used}\times 100$$

### 2.4. Functionalization of with Ribose

The CHL-Cu-MOFs were further subjected to ribose coating (Scheme 1). Briefly, CHL-Cu-MOFs (36.8 mg) were dispersed in buffer of pH 4 buffer followed by the addition of 0.203 mM of solution of ribose and stirred for 2 h at 50 °C. After that, aqueous NaBH_4_ (0.5 mmol) was added to the reaction mixture and stirred for 3 days at 50 °C. Freshly prepared NaBH_4_ (0.5 mmol) were added to the reaction mixture after every 12 h.

### 2.5. Characterization

#### 2.5.1. FT-IR Analysis

FT-IR spectra of Cu-MOFs, CHL, CHL-Cu-MOFs, and R-CHL-Cu-MOFs were recorded using IR spectrophotometer (Shimadzu, Kyoto, Japan). The samples were mixed with KBr and converted into self-supporting disc by applying a high pressure of 1.38 × 10^3^ kPa. The discs of each sample were scanned in a range of 400–4000 cm^−1^ using an IR spectrophotometer.

#### 2.5.2. Determination of Size, PDI, Zeta Potential, and Surface Morphology

The synthesized samples were diluted properly and subjected for size, PDI, and zeta potential determination through a dynamic light scattering (DLS) instrument (Nano ZS90 Malvern Instruments, Worcestershire, UK) in triplicate at a scattering angle of 90° at 25 °C. Surface morphology of the prepared Cu-MOFs samples was studied using atomic force microscope (AFM, 5500, Agilent, Santa Clara, CA, USA).

#### 2.5.3. Powder XRD

Purity and crystallinity of synthesized Cu-MOFs were determined by powder XRD. For the measurement of diffraction patterns, we used an X-ray diffraction instrument (Axios Petro, PANalytical, CoKα, λ = 1.79021 Å) from 5° to 60° (2θ) with Cu-Kα irradiation.

#### 2.5.4. In Vitro Drug Release Study

In vitro dissolution study was carried out at pH 6.8 using dialysis bag of 12,000 kDa. The ribose-coated CHL-Cu-MOFs were dispersed in water and transferred in the activated dialysis membrane. The membrane was then placed in the release medium of pH 6.8 buffer and subjected for shaking at 100 G. At specific time intervals, 2 mL of dissolution medium was withdrawn to quantify the amount of released drug and refilled with fresh buffer. The samples were then studied on UV-vis spectrophotometer at 278 nm.

### 2.6. Antibacterial Assay

#### 2.6.1. Bacterial Strains

Different strains of the test microorganism were selected for antibacterial assay which included Gram-negative bacterium, *E.coli ATCC 8739* (sensitive), *E. coli ATCC* 35,218 (resistant), and *Pseudomonas aeruginosa ATCC 10145.* Bacterial strains stock culture was kept on Tryptic soya agar (Oxoid, UK) at 4 °C. The microbial strain was sub-cultured on a fresh appropriate agar plate for 24 h prior to antibacterial test. Inocula were prepared by transferring several single colonies of microbes to a sterile Mueller Hinton broth. The microbial cell suspension was mixed to homogeneity to give a final density of 5 × 10^5^ cfu/mL and these were confirmed by viable counts. The infective dose of most microorganisms was 10^5^ cfu/mL.

#### 2.6.2. Microplate Assay of Minimum Inhibitory Concentration (MIC)

The minimum inhibitory concentration of prepared samples was determined by using tetrazolium microplate assay according to the previously reported method [44] and the assay was carried out on a 96-well clear microtiter plate. Each well was seeded with freshly harvested cell suspensions of *E. coli* (sensitive/resistant) and *P. aeruginosa* strains at 5 × 10^5^ cfu/mL. Different concentrations were prepared in Muller Hinton broth ranging from 2.5 to 250 µg of CHL, Cu-MOF, and CHL-Cu-MOFs. This wider range of concentrations was selected so the minimum possible MIC can be determined for any sample. Each concentration (200 µL) was added in triplicate wells and the plates were incubated for 24 h at 37 °C ± 0.5. After that, 50 µL of 3-(4,5-dimethylthiazol-2-yl)-2, 5-diphenyltetrazolium bromide MTT (0.2 mg/mL) was added in each well followed by incubation at 37 °C for 30 min. As a negative control, DMSO was used while bacterial cells as positive control. The absorbance was measured at 570 nm with a reference wavelength of 650 nm by adding DMSO on a spectrophotometer and the percentage reduction of the dye (indicating the bacterial growth inhibition) was calculated while using values of optical density (O.D) by using Equation (2) [45].

(2) $$\mathrm{IC}50=\frac{O.D.\;\mathrm{in}\;\mathrm{Control}-O.D.\;\mathrm{of}\;\mathrm{test}\;}{O.D.\;\mathrm{in}\;\mathrm{control}}\times 100$$

#### 2.6.3. Determination of Minimum Biofilm Inhibitory Concentration (MBIC)

Antibiofilm activity of CHL, Cu-MOFs, and CHL-Cu-MOFs were determined against *E. coli* (sensitive/resistant) and *P. aeruginosa* strains using the microtiter plate method. The compounds were diluted as mentioned above in a 96-well flat bottom plate (Corning, Glendale, AZ, USA). An inoculum containing 5 × 10^5^ CFU ml^−1^ bacteria was inoculated in each well except the broth control. Plates were incubated overnight at 37 °C and followed by staining to allow the biofilm formation [32]. Plates were then washed with distilled water thrice to remove planktonic cells followed by staining with 0.1% (*w*/*v*) crystal violet for 20 min. Stained plates were rewashed and retained crystal violet was dissolved in 30% (*v*/*v*) glacial acetic acid. Absorbance was then measured at 590 nm using microplate reader (Tecan, Chapel Hill, NC, USA). Percent biofilm inhibition was calculated by using Equation (3):

(3) $$\%\;\mathrm{biofilm}\;\mathrm{inhibition}\;=\frac{O.D.\;\mathrm{in}\;\mathrm{Control}-O.D.\;\mathrm{of}\;\mathrm{test}\;}{O.D.\;\mathrm{in}\;\mathrm{control}}\;\times \;100$$

#### 2.6.4. Surface Morphological Analysis

Morphological changes were studied by AFM and SEM analysis. Selected resistant and sensitive strains of *E. coli* and *P. aeruginosa* were harvested 37 °C in TSA for 24 h. A freshly cleaved mica slide was loaded with 10 μL of poly-lysine and allowed to dry at room temperature. The slides were then loaded with a drop of diluted cultures of bacterial strain (10^5^ cfu), dried, and analyzed for morphological investigations using AFM. Similarly, the cells incubated with test samples were used for the comparative morphological study. Samples (5–10 μL) at their MIC were withdrawn from microtiter plate and applied to poly-lysine loaded mica slides, dried at room temperature. The slides were then analyzed on AFM for morphological investigations.

SEM investigation was carried out by incubating the samples at 37 °C for 1 h by using their respective MBIC values. The cells were then fixed for 2 h with glutaraldehyde solution (2%) at 4 °C and dehydrated in a grade series of alcohol. The samples were then coated by gold coater and observed with SEM.

### 2.7. Statistical Analysis

All the experiments were carried out in triplicate form and results were expressed as mean ± SEM.

## 3. Results and Discussion

### 3.1. FT-IR Analysis

**Figure 1:**
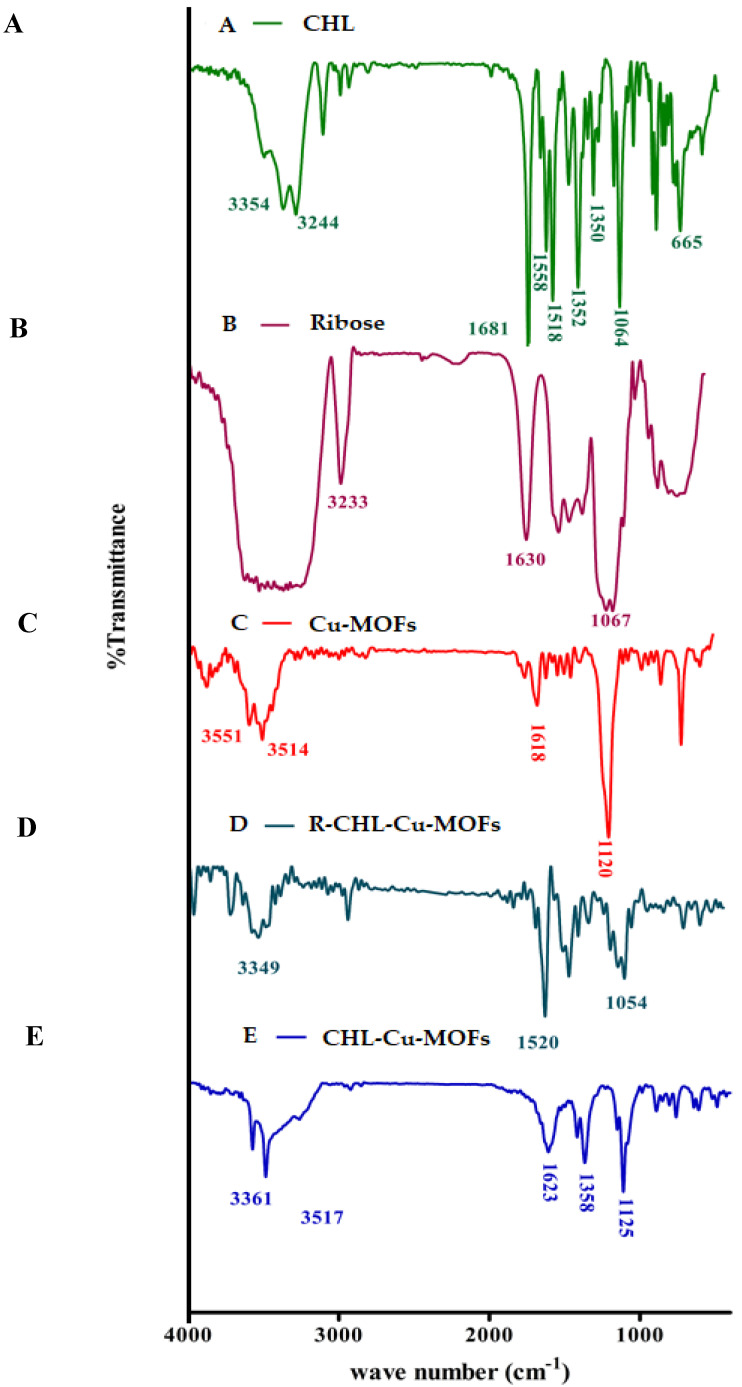
FTIR spectra of CHL (**A**), ribose (**B**), Cu-MOFs (**C**), R-CHL-Cu-MOFs (**D**), and CHL-Cu-MOFs (**E**).

FT-IR spectrum of CHL revealed characteristic peak at 3352–3246 cm^−1^ (OH and NH stretching). C=O stretching appeared at 1681 cm^−1^ while C=C stretching observed at 1558 cm^−1^. Furthermore, the prominent NO_2_ vibration appeared at 1518 cm^−1^ with C-Cl stretching at 665 cm^−1^ (Figure 1A) [46]. Cu-MOFs showed characteristic peaks at 3551 and 3514 cm^−1^ for NH stretching, and 1618 cm^−1^ for C=O stretching with a sharp peak at 1120 cm^−1^ (Figure 1B). After CHL encapsulation in Cu-MOFs, the characteristic peak of Cu-MOFs slightly shifted with a change in appearance at 3361 and 2517 cm^−1^ which shows the interaction of respective functional groups of Cu-MOFs with CHL (Figure 1C). The characteristic peaks of ribose appeared in the region of 3600–3200 cm^−1^ showing the O-H stretching, while C-O-C absorption appeared at 1067 cm^−1^ (Figure 1D). After the coating of ribose on the CHL-Cu-MOFs, the peaks of the region of 3600–3200 cm^−1^ was shifted to 3349 cm^−1^ showing the significant involvement of OH group. Furthermore, the NH stretching of CHL-Cu-MOFs disappeared in R-CHL-Cu-MOFs, suggesting the involvement of amino group to interact with ribose and the resulting new functionality of C=N appeared at 1520 cm^−1^ (Figure 1E), showing the successful coating of ribose on CHL-Cu-MOFs.

### 3.2. Determination of Size, PDI, Zeta Potential, and Surface Morphology

**Figure 2:**
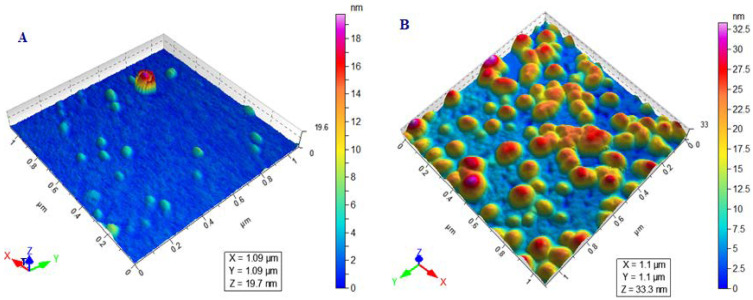
AFM images showing the spherical shape morphology of Cu-MOFs (**A**) and R-CHL-Cu-MOFs (**B**).

In drug delivery systems, particle size is considered as the most important parameter because it is significantly affects the therapeutic performance and physical stability of the loaded drug. MOFs are the porous nanocarriers and their size is mostly controlled by the method of preparation and the use of solvents during their preparation, i.e., the larger particle size is obtained by the synthesis in water and methanol while the smaller size is obtained by using DMF as a solvent. The reason behind this is the greater solubility of cross-linker in DMF as compared to water and methanol [47]. The synthesized Cu-MOFs revealed 394.10 ± 16.45 nm particle size with the PDI of 0.50 ± 0.07. The increase in particle size was observed after encapsulation of CHL and modification with ribose which is equivalent to 412.52 ± 19.84 and 562.84 ± 13.42 nm, respectively, with an almost similar PDI value, i.e., 0.47 ± 0.05 for CHL-Cu-MOFs, while it was increased to 1.00 ± 0.08 for R-CHL-Cu-MOFs as shown in Table 1. On the other hand, the zeta potential is another important factor associated with the stability of particles and helps to remain suspended. The negative zeta potential was observed for the synthesized Cu-MOFs and the surface negativity is because of the presence of aminoterephthalic acid in the Cu-MOFs structure. Interestingly, the surface negativity was unaffected by the CHL loading and ribose coating as shown in Table 1. Furthermore, the synthesized Cu-MOFs was spherical in shape as authenticated by the AFM images described in Figure 2. The figure also revealed the increase in particle size upon CHL loading and ribose coating which is consistent with the DLS results.

### 3.3. Drug Encapsulation Efficiency

The entrapment of drugs in the designed drug delivery carrier ensures the delivery of the therapeutics at the target site with enhanced efficacy. Basically, the loading of the drug candidate in the MOF structure is strictly related to their surface area, as well as large tunnel and cages as the entrapment is mostly actually occurred in MOFs cages and tunnels [48]. In the designed Cu-MOFs, 45.23 ± 2.22% of CHL was loaded. The entrapment mechanism in the Cu-MOFs may involve the inclusion of CHL in their pores or adsorption on their surfaces by secondary interactions (hydrogen bonding and pi–pi stacking). The entrapment efficiency of Cu-MOFs was decreased slightly after their coating with ribose (Table 1), which may be due to the removal of CHL which were loosely bounded with Cu-MOFs during the process of ribose conjugation.

### 3.4. Powder XRD

**Figure 3:**
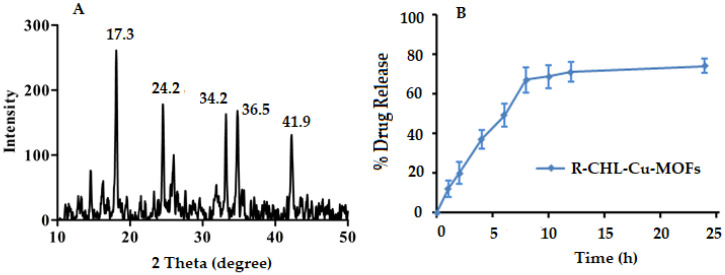
Powder XRD pattern of Cu-MOFs (**A**), and in vitro dissolution profile of ribose-coated CHL-Cu-MOFs (**B**).

Powder XRD is one of the best characterization techniques which is used to study the structural feature of nanomaterials. The nanomaterial showed characteristics which are based on microstructure length comparative with critical length scale, hence providing unique mechanical, optical, and electronic features. XRD patterns contain the basic evidence regarding phase composition, crystal size, and also possess the information from lattice strain to crystallographic orientation [49]. Thus, the synthesized Cu-MOFs were evaluated for their phase purity and crystallinity using powder XRD. The XRD pattern of Cu-MOFs is shown in Figure 3A, which exhibited the main crystalline peaks at 17.3° and 24.2° which was also observed in the XRD pattern described in the literature [50] with some additional peaks 34.2°, 36.5°, and 41.9°. The clear XRD pattern with sharp peaks provides significant authentication for the synthesis of crystalline Cu-MOFs.

### 3.5. In Vitro Release Study

The in vitro dissolution study was carried out at physiological pH of 6.8 and the release pattern is given in Figure 3B. The synthesized ribose-coated CHL-Cu-MOFs showed 12.24 ± 4.30% release of CHL at initial 1 h which was capable to release CHL with respect to time and reached a maximum release of almost 67.42 ± 6.41% at 8 h of the experiment. After that, the CHL release from the MOFs became sustained until 24 h with a slight increment to 74.32 ± 3.80%.

### 3.6. Antibacterial Assay

#### 3.6.1. Determination of MIC Value

**Figure 4:**
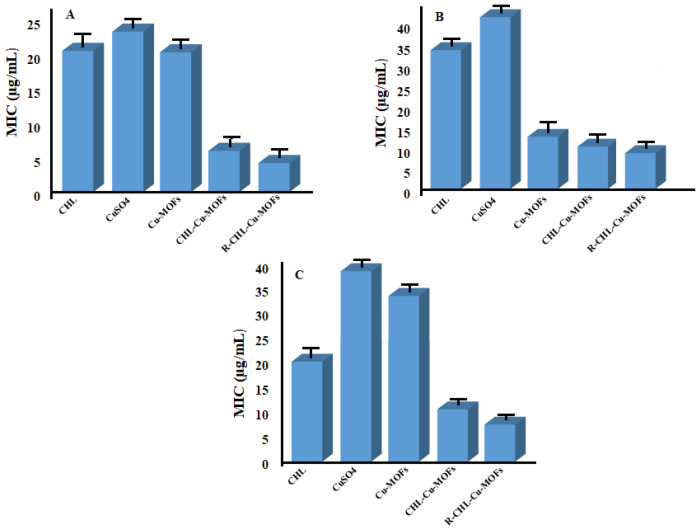
MIC of CHL, CuSO4, Cu-MOFs, CHL-Cu-MOFs, and R-CHL-Cu-MOFs against sensitive *E. coli* (**A**), resistant *E. coli* (**B**), and *P. aeruginosa* (**C**).

The tetrazolium microplate assay was used to determine the MIC value of CHL. The MIC value of CHL and CuSO_4_ against sensitive *E. coli (E. coli* (S)) was observed as 20.44 ± 2.4 and 24.86 ± 2.18 µg/mL, respectively, while for the resistant *E. coli (E. coli* (R) strain, it was 33.46 ± 3.16 and 44.98 ± 2.86 µg/mL, respectively. The MIC value was decreased (5.96 ± 2.80 µg/mL) for CHL-loaded CHL-Cu-MOFs against *E. coli* (S), while it was 10.24 ± 1.64 µg/mL against the *E. coli* (R) strain. The MIC was further decreased to 4.20 ±1.82 µg/mL for R-CHL-Cu-MOFs against *E. coli* (S) while it was 8.60 ± 3.22 µg/mL for *E. coli* (R) strains. In the case of *P. aeruginosa,* CHL showed an MIC value around 20.16 ± 2.22 µg/mL, while the other control, CuSO_4_, exhibited 39.80 ± 23.22 µg/mL. The MIC value was decreased for CHL-Cu-MOFs and R-CHL-Cu-MOFs which is equivalent to 10.54 ± 1.65 and 7.46 ± 2.17 µg/mL, respectively (Figure 4). The results of the study revealed that the bactericidal potential of CHL increased significantly upon encapsulation in ribose-coated Cu-MOFs. This is because of the interaction of ribose with lectins present on the bacterial cell surface for enhancing the antibacterial activity of CHL [51].

Similarly, IC_50_ values of CHL against *E. coli* (S) and *E. coli* (R) cells were found to be 48.12 ± 2.14 and 51.86 ± 1.98 µg/mL, respectively. CuSO_4_ showed comparatively higher IC_50_ against *E. coli* (S) and *E. coli* (R) which is equivalent to 53.12 ± 3.34 and 59.22 ± 2.84 µg/mL, respectively, while the IC_50_ value of Cu-MOFs was observed as 50.22 ± 1.43 µg/mL against both the strains. CHL-Cu-MOFs showed a decreased IC_50_ value up to 41.21 ± 2.86 µg/mL while it was further reduced to 39.0 ± 3.10 µg/mL against the sensitive strain after ribose coating. For *E. coli* (R), CHL-Cu-MOFs showed a decreased IC_50_ value of 45.22 ± 2.98 µg/mL while it was further decreased to 43 µg/mL upon coating with ribose. IC_50_ of CHL and Cu-MOFs against *P. aeruginosa* was found to be 75.04 ± 1.24 and 80.46 ± 3.14 µg/mL, respectively, while CuSO_4_ exhibited the IC_50_ 92.42 ± 2.14 µg/mL. CHL upon encapsulation in Cu-MOFs significantly reduced the IC_50_ up until 50.06 ± 2.18 µg/mL which was subsequently decreased to 44 µg/mL after being coated with ribose. Results revealed that ribose coating on CHL-Cu-MOFs significantly reduced the IC_50_ value of CHL against all tested bacterial strains.

**Figure 5:**
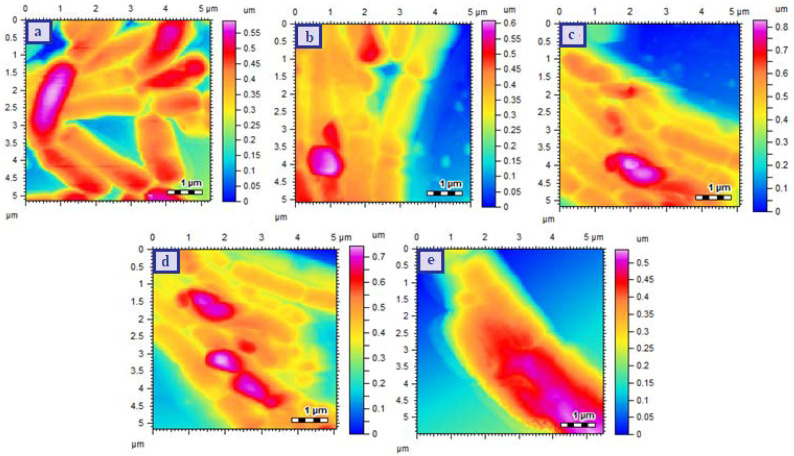
AFM images of *E. coli* (S) Control (**a**), CHL treated (**b**), Cu-MOFs treated (**c**), CHL-Cu-MOFs treated (**d**) and R-CHL-Cu-MOFs treated (**e**). The images were takne taken after exposing the bacteria to the minimum inhibitory concentrations of the samples.

These results were also confirmed by AFM showing the morphological changes of bacterial strains after treatment with test samples. The AFM images of bacterial strains are given in Figure 5. The control of *E. coli* (S) strain was observed as rods (Figure 5a) and the bacterial strains maintained their morphological characteristics as no visible change was observed after treatment with CHL and Cu-MOFs (Figure 5b,c respectively). The bacterial morphology was some-how disturbed upon treatment with CHL-Cu-MOFs (Figure 5d) and become completely distorted as no proper rod was observed when treated with R-CHL-Cu-MOFs (Figure 5e).

**Figure 6:**
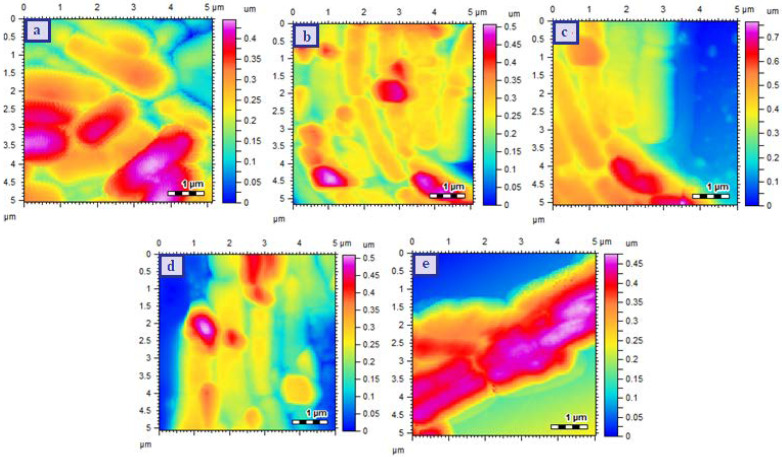
AFM images of *E.coli* (resistant) Control (**a**), CHL treated (**b**), Cu-MOFs treated (**c**), CHL-Cu-MOFs treated (**d**), and R-CHL-Cu-MOFs treated (**e**). The images were takne taken after exposing the bacteria to the minimum inhibitory concentrations of the samples.

The AFM images of *E. coli* (R) strains are provided in Figure 6 and the results revealed the proper rod shaped cellular structure for *E. coli* (R) control strain (Figure 6a). Treatment with CHL slightly affected the bacterial morphology as some of the cells was damaged and lost their original morphological feature (Figure 6b) but the cells were unaffected upon incubation with Cu-MOFs (Figure 6c). The damage in bacterial cell was increased upon treatment with CHL-Cu-MOFs and completely melted material was observed when treated with R-CHL-Cu-MOFs (Figure 6d,e respectively).

**Figure 7:**
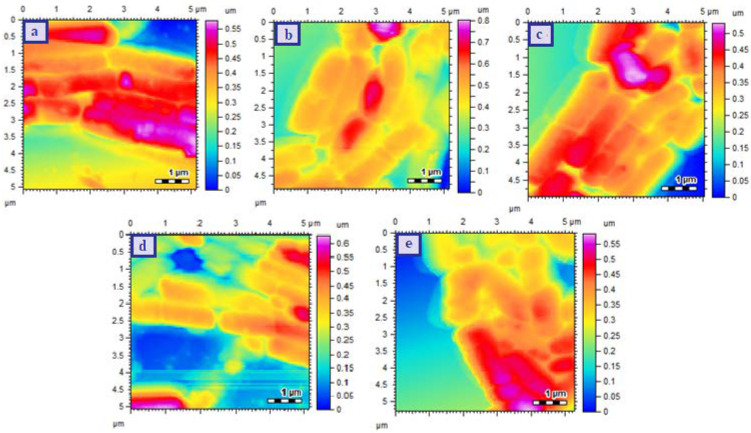
AFM images of *P. aeruginosa* Control (**a**), CHL treated (**b**), Cu-MOFs treated (**c**), CHL-Cu-MOFs treated (**d**), and R-CHL-Cu-MOFs treated (**e**). The images were takne taken after exposing the bacteria to the minimum inhibitory concentrations of the samples.

The morphological changes of *P. aeruginosa* are shown in Figure 7. The control *P. aeruginosa* showed rod shaped structure which was arranged in a proper chain (Figure 7a). The cellular morphology was maintained when treated with CHL but the chain arrangement was disturbed while neither arrangement nor structure was affected upon treatment with Cu-MOFs (Figure 7b,c respectively). The bacterial cells treated with CHL-Cu-MOFs showed significant disturbance in arrangement with slight effect on morphology (Figure 7d). The more prominent effect was observed for R-CHL-Cu-MOFs treated bacterial cells as the proper rods and their arrangements were completely disappeared (Figure 7e). Hence, for all the bacterial strains used in this study, the significant effect was observed for the R-CHL-Cu-MOFs and it is because of the fact that bacterial surface possesses sugar binding lectins which are capable to interact with ribose [39,40]. With the help of ribose-lectin interaction, the cells were eventually interacted with CHL and become destroyed via action of CHL.

#### 3.6.2. Determination of MBIC Value

**Figure 8:**
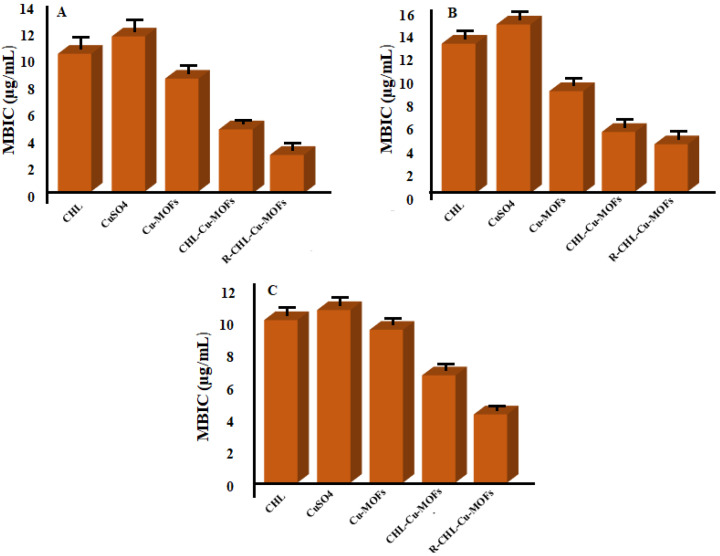
MBIC values of CHL, CuSO_4_, CHL-Cu-MOFs, and R-CHL-Cu-MOFs against sensitive *E. coli* (**A**), resistant *E. coli* (**B**), and *P. aeruginosa* (**C**).

Biofilms are the major mode of microbial life [52] which help bacterial cells to survive in unfavorable condition [53]. Biofilms are also responsible for the emergence of bacterial resistance against antibiotics [54]. Hence the synthesized Cu-MOFs were also investigated for their MBIC value which was further authenticated by SEM analysis. The MBIC value of CHL for the *E.coli* (S) was reduced from 10.16 ± 3.42 µg/mL to 5.14 ± 2.75 µg/mL and 3.21 ± 1.98 µg/mL when encapsulated in Cu-MOFs and ribose-coated Cu-MOFs respectively. MBIC value for CuSO_4_ was found to be 12.17 ± 2.42 µg/mL. Similarly, for the resistant strain, it was reduced to 5.71 ± 2.44 µg/mL (CHL-Cu-MOFs) and 4.40 ± 1.79 (R-CHL-Cu-MOFs). While the MBIC of CHL and CuSO_4_ was found to be 12.89 ± 1.12 µg/mL and 14.5 ± 1.42 µg/mL, respectively. On the other bacterial strain *P. aeruginosa,* the MBIC value of simple CHL was 20.19 ± 2.79 µg/mL which was reduced to 6.60 ± 1.87 µg/mL for CHL-Cu-MOFs and 4.10 ± 1.22 µg/mL for R-CHL-Cu-MOFs, while the MBIC value for the CuSO_4_ as observed as 11.98 ± 2.24 µg/mL (Figure 8). The significantly increased activity of CHL after being encapsulated in ribose-coated Cu-MOFs may be associated with the resemblance of ribose with the AI-2 which exhibits the competing behavior with AI-2 and acts as an effective QSI for the inhibition of bacterial biofilm formation [35].

**Figure 9:**
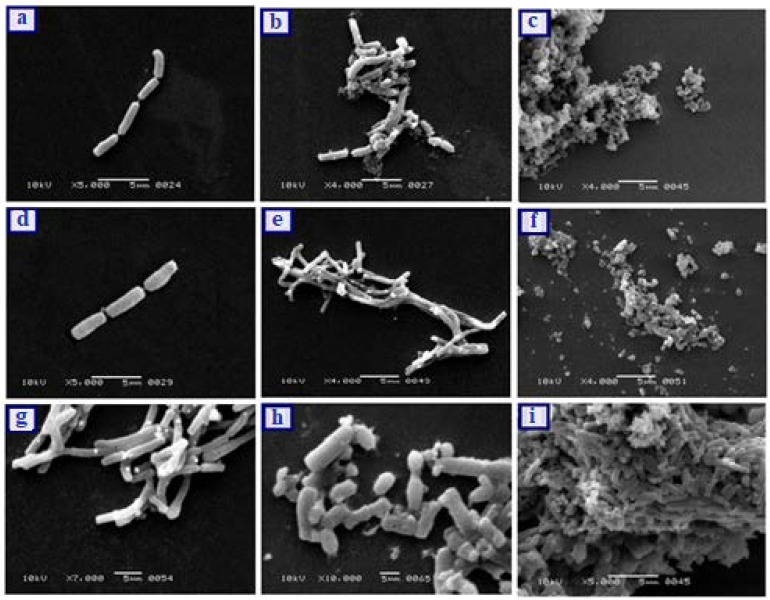
SEM of *E. coli* (S) control (**a**), *E. coli* (S) biofilm treated CHL-Cu-MOFs (**b**), *E. coli* (S) biofilm treated R-CHL-Cu-MOF (**c**), *E. coli* (R) control (**d**), *E. coli* (R) biofilm treated with CHL-Cu-MOFs (**e**), *E. coli* (R) biofilm treated with R-CHL-Cu-MOFs (**f**), *P. aeruginosa* control (**g**), *P. aeruginosa* biofilm treated with CHL-Cu-MOFs (**h**), and *P. aeruginosa* biofilm treated CHL-Cu-MOF (**i**). The images were taken after exposing the bacteria to the minimum inhibitory concentrations of the samples.

The results of MBIC study were also confirmed by the investigation of morphological changes of bacterial strains after being treated with test samples. The SEM images of all the bacterial strains are presented in Figure 9. The SEM image of *E. coli* (S) showed the smooth rod-shaped structure in chain arrangement while when treated with CHL-Cu-MOFs, the smoothness of bacterial cell became disturbed and some of the cells separated from their regular arrangement (Figure 9a,b respectively). The cells were completely destroyed when treated with R-CHL-Cu-MOFs (Figure 9c). The *E. coli* (R) was also observed as rods arranged in chain (Figure 9d) and the morphology of bacterial cell was unaffected by the treatment with CHL-Cu-MOFs, but became completely distorted when treated with R-CHL-Cu-MOFs (Figure 9e,f respectively). The SEM image of the *P. aeruginosa* control revealed the smooth rod-shaped morphology and the smoothness of the cells was affected by the influence of CHL-Cu-MOFs (Figure 9g,h respectively). The treatment with R-CHL-Cu-MOFs increased the roughness of the structure and completely destroyed most of the bacterial structure (Figure 9i).

## 4. Conclusions

This study reported the synthesis of ribose-coated Cu-MOFs for the enhanced antibacterial potential of CHL. The synthesized Cu-MOFs were capable to encapsulate the increased amount of CHL and significantly increase its potential against bacterial population. The ribose-coated CHL-Cu-MOFs showed the promising activity against *E. coli* sensitive and resistant strains as well as MDR pathogen, *P. aeruginosa*, and significantly reduced their MIC and MBIC values. The results were further authenticated by their morphological investigation using SEM and AFM which showed the complete distortion of bacterial cells upon treatment with R-CHL-Cu-MOFs. The results of the study revealed that ribose-coated Cu-MOFs can significantly overcome the MDR of pathogenic bacteria and represent a promising antibacterial candidate for preclinical application to treat various bacterial infections.

## Figures and Tables

**Table 1:** Size, PDI, zeta potential, and encapsulation efficiency of synthesized Cu-MOFs.

Samples	Size (nm)	PDI	Zeta Potential (mV)	%EE
Cu-MOFs	394.10 ± 16.45	0.50 ± 0.07	−11.42 ± 1.32	-
CHL-Cu-MOFs	412.52 ± 19.84	0.47 ± 0.05	−12.51 ± 1.84	45.23 ± 2.22%
R-CHL-Cu-MOFs	562.84 ± 13.42	1.00 ± 0.08	−10.53 ± 0.98	44.15 ± 3.15%

## Data Availability

The data supporting this study are available from corresponding author upon reasonable request.
